# Therapieoptionen des Lymphödems

**DOI:** 10.1007/s00117-025-01441-1

**Published:** 2025-04-22

**Authors:** Christian Dirk Taeger, Claus Christian Pieper, Daniel Schiltz

**Affiliations:** 1 Plastische Chirurgie & Ästhetik an der Isar, Widenmayerstraße 16, 80538 München, Deutschland; 2 Sektion für minimal invasive Lymphgefäßtherapie, Klinik für Diagnostische und Interventionelle Radiologie, Universitätsklinikum Bonn, Venusberg-Campus 1, 53127 Bonn, Deutschland; 3 Zentrum für seltene angeborene Lymphgefäßerkrankungen, Zentrum für seltene Erkrankungen Bonn (ZSEB), Universitätsklinik Bonn, Venusberg-Campus 1, 53127 Bonn, Deutschland; 4 Klinik für Plastische und Ästhetische Chirurgie, Handchirurgie, Helios Klinikum Emil von Behring, 14163 Berlin, Deutschland

**Keywords:** Lymphchirurgie, Lymphovenöse Anastomose, VLNT, Mikrochirurgie, Supermikrochirurgie, Lymphatic surgery, Lymphovenous anastomosis, Vascularized lymph node transfer, Microsurgery, Supermicrosurgery

## Abstract

**Hintergrund:**

Das Lymphödem ist eine chronisch progrediente Erkrankung, die unbehandelt zu einer massiven Einschränkung der Lebensqualität und Folgeerkrankungen wie rezidivierenden Erysipele führen kann. Die rein konservative Therapie galt lange Zeit als einzige Behandlungsmöglichkeit des primären und sekundären chronischen Lymphödems der oberen bzw. unteren Extremität. Lediglich bei sehr ausgeprägten Spätformen kamen u. a. resezierende Verfahren, wie die Liposuktion oder Gewebeabtragungen als weitere Therapieoptionen in Frage. Durch eine – teils sprunghafte – Weiterentwicklung diagnostischer und mikrochirurgischer Verfahren steht nun eine Reihe moderner rekonstruktiver Therapieverfahren zur Verfügung, mit deren Hilfe die Erkrankungsschwere in Händen versierter Mikrochirurgen in vielen Fällen signifikant gelindert werden kann.

**Ziel der Arbeit:**

Ziel dieses Artikels ist es, die modernen Therapieoptionen des Lymphödems nach aktuellem Stand der Wissenschaft praxisorientiert dazulegen und einen Leitfaden zur optimalen Behandlung betroffener Patienten mitzugeben.

**Diskussion:**

Neben einer unverzichtbaren konservativen Therapie führen moderne mikrochirurgische Verfahren in vielen Fällen zu einer erfolgreichen und dauerhaften Symptomlinderung und sollten frühzeitig in Erwägung gezogen werden. Die präoperative Diagnostik ist dabei für die Wahl der richtigen Therapie und deren Erfolg unerlässlich.

## Abstract (en)

**Background:**

Lymphoedema is a chronically progressive disease which, if left untreated, can lead to a massive reduction in quality of life and secondary diseases such as recurrent erysipelas. For a long time, purely conservative therapy was considered the only treatment option for primary or secondary chronic lymphedema of the upper or lower extremities. Only in very pronounced late forms were resection procedures such as liposuction or tissue removal considered as further treatment options. A series of modern reconstructive therapy procedures are now available thanks to the—in some cases rapid—further development of diagnostic and microsurgical procedures, which in many cases can significantly alleviate the severity of the disease in the hands of experienced microsurgeons.

**Aim:**

The aim of this article is to present the modern treatment options for lymphedema according to the current state of scientific knowledge in a practice-oriented manner and to provide guidelines for the optimal treatment of affected patients.

**Conclusion:**

In addition to indispensable conservative therapy, modern microsurgical procedures can lead to successful and lasting symptom relief in mandy cases and should be considered at an early stage. Preoperative diagnostics are essential for choosing the right treatment and its success.

Das chronische Lymphödem, sei es primär, also anlagebedingt, oder sekundär nach beispielsweise Tumoroperationen oder Unfällen, ist eine Lymphabflussstörung, die in den meisten Fällen die obere oder untere Extremität betrifft [9] und stellt eine enorme Belastung für die Betroffenen, aber auch für unser Gesundheitssystem dar [17]. Diese Abflussstörung der Lymphe führt zu einer Erhöhung des intravasalen Drucks im Lymphgefäßsystem. Vergleichbar mit der arteriellen Hypertonie schädigt beim Lymphödem der erhöhte intravasale Druck die Lymphgefäße. Nach einer initialen Dilatation der Lymphgefäße kommt es zur Inflammation und in der Folge zur Sklerosierung und zum Verschluss der Lymphbahnen [16]. Die Lymphlast kann folglich nicht mehr abtransportiert werden, und es kommt zur Lymphostase. Diese Flüssigkeitsansammlung lässt die betroffene Extremität an Umfang zunehmen. Initial handelt es sich dabei um ein *dellbares Ödem*, das heißt, dass nach längerer Druckapplikation mittels Finger, beispielsweise prätibial oder am Handrücken des Betroffenen, eine *stehende Delle* für kurze Zeit sichtbar bleibt (Abb. 1).

**Abb. 1:**
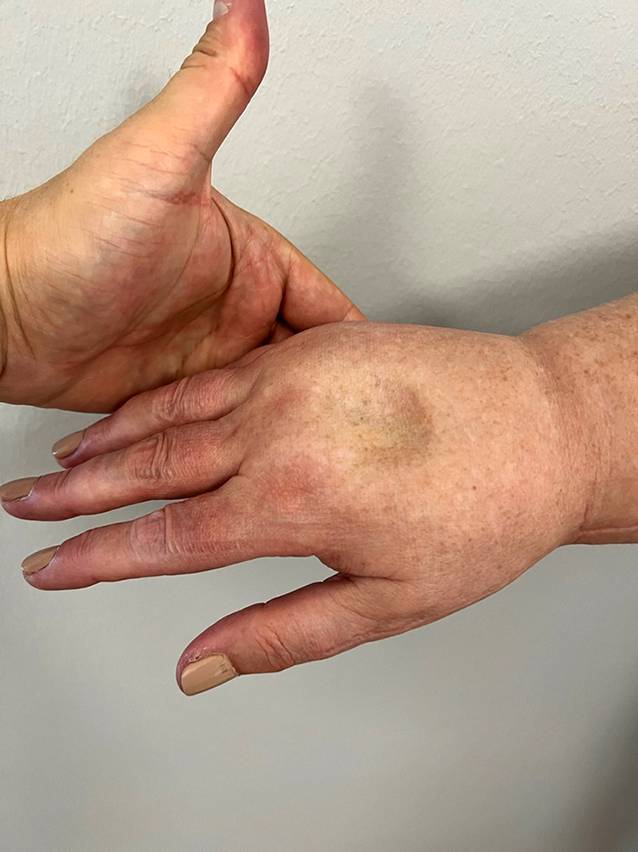
*Stehende Delle* bei einer Patientin mit sekundärem Lymphödem Stadium II

Ein über einen längeren Zeitraum bestehendes Lymphödem führt nicht selten zur Verhärtung, die Dellbarkeit nimmt ab. Meist wird dieser Prozess pauschal als Fibrosierung bezeichnet [24]. Im Rahmen der Fibrosierung kommt es zudem zu einer signifikanten Einwanderung von Fettzellen, wodurch im Endstadium im wesentlichen die Umfangszunahme der betroffenen Extremität zu erklären ist [4]. Dieser Umbau des Bindegewebes (Fibrosierung und Einwanderung von Fettzellen) ist ohne invasive Maßnahmen nur schwer wieder rückgängig zu machen [2]. In Abb. 2 ist dieser Teufelskreislauf schematisch dargestellt.

**Abb. 2:**
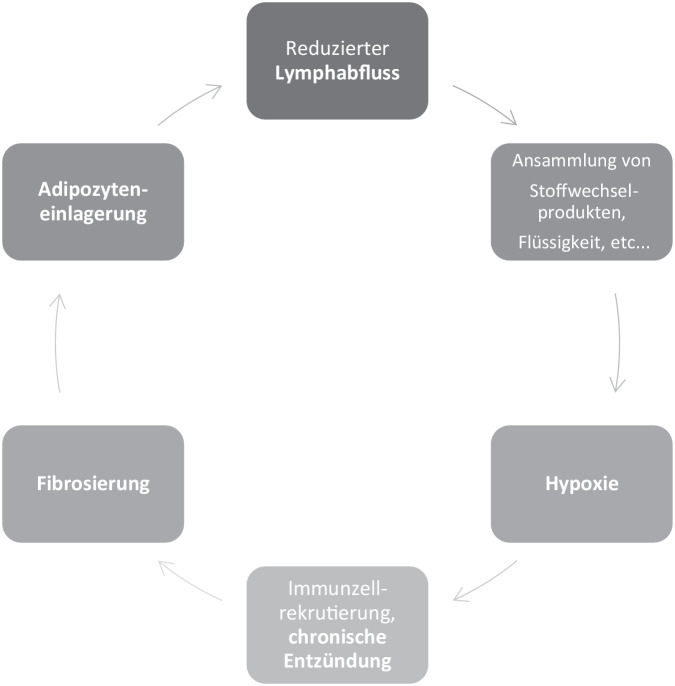
Schematische Darstellung des Teufelskreises bei unbehandeltem chronischem Lymphödem. (Aus [2])

Moderne mikrochirurgische Rekonstruktionsverfahren, bei denen der Lymphabfluss rekonstruiert werden soll, sind also in erster Linie auf Stadien ausgerichtet, bei denen dieser Umbau noch nicht vollständig abgeschlossen ist, also noch das *dellbare Lymphödem* vorliegt (bis Stadium II). Bedauerlicherweise werden weiterhin vornehmlich Patienten mit weit fortgeschrittenen Stadien (Stadium II bis III) zur operativen Therapie vorgestellt, getreu dem Motto: „Erst wenn die konservative Therapie nicht mehr hilft, sollte die Chirurgie zum Einsatz kommen.“ Zu diesem Zeitpunkt kann die rekonstruktive Chirurgie oft allerdings nur noch geringe Verbesserungen erzielen.

Die relativ junge Disziplin der sog. *Lymphchirurgie* kennt viele Facetten, es wurden in der Vergangenheit verschiedenste Verfahren entwickelt und auch wieder verworfen. Die zum jetzigen Zeitpunkt am häufigsten durchgeführten Verfahren sind die Anlage von lymphovenösen Anastomosen (LVA; [5]) und der freie vaskularisierte Lymphknotentransfer (VLNT; [14]). Daher sollen diese beiden Verfahren im Folgenden näher dargestellt werden. Zudem soll im Anschluss das Vorgehen bei weiter fortgeschrittenen Befunden beschrieben werden.

## Diagnostik

Zur präzisen Diagnosestellung stehen neben der Basisdiagnostik, bestehend aus Anamnese und klinischer Untersuchung, weiterführenden bildgebende Methoden zur Verfügung.

## Bildgebung

### Lymphszintigraphie

Bei der Lymphszintigraphie werden radioaktive Tracer in die zu untersuchende Extremität distal subkutan injiziert und gemeinsam mit der Lymphe abtransportiert. Mittels Gamma-Kamera kann der Tracer und damit der Lymphabstrom sichtbar gemacht und beurteilt werden. Als Planungstool für die Lymphchirurgie ist diese Methode allerdings nicht präzise genug, weshalb hier nicht weiter auf diese Technik eingegangen wird.

### Indocyaningrün-Fluoreszenzbildangiographie

**Abb. 3:**
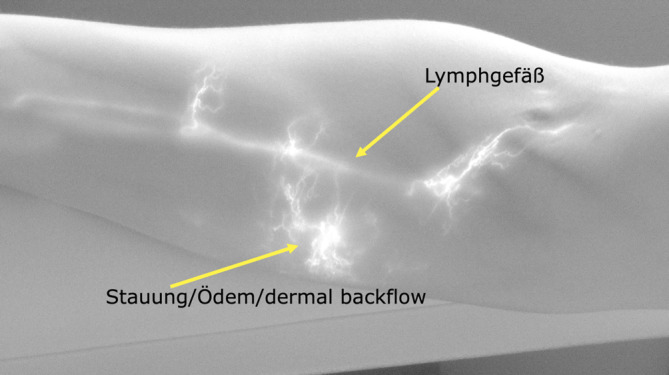
Bild eines Lymphödems Stadium 0 am Arm, dargestellt durch eine Fluoreszenzbildkamera nach Injektion von Indocyaningrün (ICG). Das Stadium 0 ist klinisch inapparent, Patienten leiden jedoch teils unter Beschwerden in den Arealen mit Ödem

Die am häufigsten durchgeführte Bildgebung im Rahmen der Lymphödemdiagnostik ist die Indocyaningrün-Fluoreszenzbildangiographie [11]. Hierbei wird der Fluoreszenzfarbstoff Indocyaningrün (ICG) in den Interdigitalraum und teils an weiteren Stellen der betroffenen Extremität injiziert [6]. Der Farbstoff wird dann von Lymphkollektoren (sofern vorhanden und funktionsfähig) aufgenommen, sodass die Bahnen bis zu einer gewissen Tiefe (abhängig von der verwendeten Kamera, bis zu 2 cm Eindringtiefe) an der Hautoberfläche mittels Fluoreszenzbildkamera dargestellt und markiert werden können (sog. „lymphatic mapping“, siehe Abb. 3). Dabei bestehen bei den Fluoreszenzbildkameras bzgl. der visualisierbaren Gewebetiefe und somit Detektionsraten enorme Qualitätsunterschiede der einzelnen Hersteller. Obwohl im Laufe der letzten Jahre bereits deutlich bessere Systeme zur Bildgebung hergestellt werden konnten, werden in aktuellen Studien zum Vergleich verschiedener bildgebender Verfahren im Bereich der Lymphologie nicht selten technisch veraltete Geräte miteinander verglichen.

In vielen Fällen kommt es im Verlauf eines Lymphkollektors dann zu einem sog. „dermal backflow“ [26], bei dem sich die eingefärbte Lymphe in dem umliegenden Gewebe verteilt. Oft zeigt sich der Schaden der Lymphbahnen an den Extremitäten beim sekundären Lymphödem von proximal nach distal fortschreitend, beim primären Lymphödem ist dies in der Regel umgekehrt [7]. Trotzdem ist das Lymphödem bei beiden Formen in den meisten Fällen distal aufgrund der Wassersäule stärker ausgeprägt als proximal. Der erwähnte „dermal backflow“ kann sich nach Injektion teils sehr schnell ausbreiten und auch intakte Lymphkollektoren überdecken. Daher ist es ratsam, die Lymphbahnen direkt nach Injektion zu markieren. Bei sehr ausgeprägten Schäden im Bereich der Lymphbahnen kann es allerdings auch vorkommen, dass das ICG langsam weiter nach proximal fließt und sich dann teils weitere Lymphbahnen abgrenzen lassen. Die Autoren raten daher zu einer Injektion und Bildgebung am Vortag des geplanten Eingriffs und einer erneuten Bildgebung am Operationstag. Dies spielt insbesondere beim primären Lymphödem eine besondere Rolle, da hier oft distale Lymphbahnen geschädigt sind und der Abtransport nach proximal deutlich mehr Zeit in Anspruch nehmen kann. An beiden Untersuchungszeitpunkten sollten sichtbare Lymphbahnen auf der Hautoberfläche markiert werden. Nach einer gewissen Einwirkzeit zeigen sich dann typische Verteilungsmuster des Farbstoffs, die Rückschlüsse auf den Fortschritt des Schadens im Bereich der Lymphbahnen zulassen [26] und dokumentiert werden sollten. Diese reichen von einer Darstellung lineare Lymphkollektoren über ein sog. „stardust“ bis hin zum „diffusen dermal backflow“. Es sei darauf hingewiesen, dass es sich bei der Verwendung von ICG um einen „off-label use“ handelt und über etwaige Risiken aufgeklärt werden muss.

### MR-Lymphangiographie

**Abb. 4:**
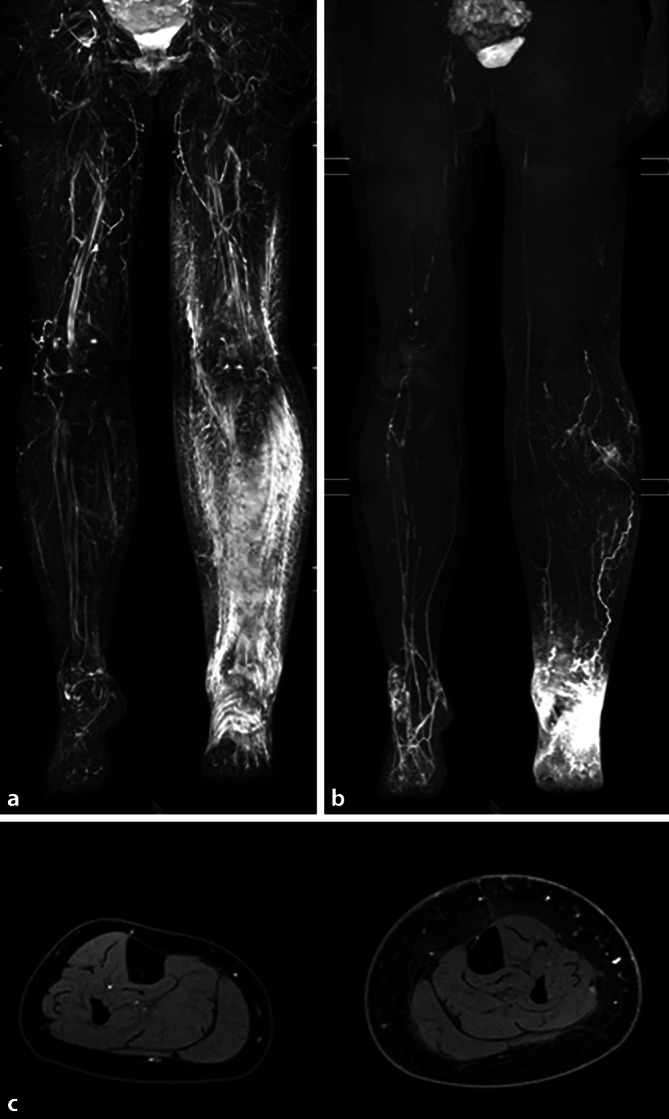
Typisches MR-Lymphangiogramm einer Patientin mit sekundärem Lymphödem. **a** Maximum-Intensitätsprojektion (MIP) einer fettunterdrückten T2-Bildgebung. **b** MIP und **c** axiale Schicht des hochaufgelösten T1-gewichteten Lymphangiogramms nach Kontrastmittelgabe. Es zeigt sich ein ausgedehntes subkutanes Ödem des linken Beines mit dilatierten und nur bis zum distalen Oberschenkel kontrastiert abgrenzbaren oberflächlichen Lymphgefäßen mit entsprechendem „dermal backflow“. Rechts erhaltener Lymphabstrom mit auch Kontrastierung tiefer, intermuskulärer Lymphgefäße

Die Lymph-MRT bietet den besonderen Vorteil, das Lymphsystem auch in tieferen Schichten und auch in seiner Gesamtheit darstellen zu können (Abb. 4; [8, 19, 20]). Die Verfügbarkeit in Deutschland ist zum gegenwärtigen Stand insbesondere aufgrund eines verhältnismäßig großen logistischen und zeitlichen Aufwandes für eine entsprechende MRT-Untersuchung jedoch noch sehr limitiert, weshalb die Indikation für eine Lymph-MRT wohlüberlegt gestellt werden sollte. Aus Sicht der Autoren ist insbesondere das primäre Lymphödem eine Indikation, da sich hier eine große Bandbreite an Anomalien im Bereich des Lymphsystems verbergen kann, die man mit der ICG-Fluoreszenzbildangiographie aufgrund der oben erwähnten geringen Eindringtiefe nicht immer vollumfänglich beurteilen kann. Beispielhaft sei hier der Fall eines primären Lymphödems genannt, bei dem lediglich die Anzahl der Lymphkollektoren reduziert ist, ihre Kontinuität jedoch vorliegt. Hier wäre die Anlage von LVAs folglich nicht ratsam. Gerade auf Höhe des Oberschenkels kann es vorkommen, dass man mit der ICG-Fluoreszenzbildangiographie aufgrund der erhöhten Gewebedicke falsche Schlüsse zieht: Wenn beispielsweise Kollektoren vom oberflächlichen in das tiefe intermuskuläre System wechseln, kann dies den Anschein haben als wären diese unterbrochen. Außerdem ist die Indikation für eine Lymph-MRT bei sehr ausgeprägten Lymphödemen gegeben, da auch hier die Schichtdicke (bereits eingewanderte Fettzellen bei Stadium II und III) eine umfassende Darstellung des Lymphsystems durch die ICG-Fluoreszenzbildangiographie nicht zulässt.

Nachteilig an der Lymph-MRT könnte sein, dass man meist nach Injektion des Kontrastmittels relativ zeitnah eine Bildgebung durchführt und ggf. sich erst später demarkierende Strukturen übersehen werden könnten, da die Kontrastmittelverteilung nach proximal noch länger benötigt hätte [21]. Auch im Rahmen der MR-Lymphangiographie ist es wichtig zu betonen, dass die Anwendung von MRT-Kontrastmittel zur Darstellung von Lymphgefäßen einen „off-label use“ darstellt und die Patienten entsprechend aufgeklärt werden müssen.

#### Weitere bildgebende Verfahren

Mittels Hochfrequenzultraschall können in versierter Hand Lymphgefäße dargestellt werden, die z. B. mittels ICG-Fluoreszenzbildangiographie oder Lymph-MRT nicht gezeigt werden können, da sie schlichtweg vom applizierten Farbstoff bzw. Kontrastmittel nicht erfasst wurden [10]. Allerdings geht hier die globale Beurteilung des Lymphabstroms verloren, was wiederum zu etwaigen Fehlentscheidungen im Rahmen der chirurgischen Therapie führen kann. Ein weiteres Verfahren ist die sog. multispektrale optoakustische Tomographie. Hierbei kann beispielsweise ICG, das vom lymphatischen System abtransportiert wird, durch Ultraschall detektiert und visuell in 3D dargestellt werden [10]. Dieses Verfahren wird aktuell in Studien näher untersucht.

## Therapieverfahren

### Konservative Therapie

Die konservative Therapie ist ein ganz wesentlicher Bestandteil einer dauerhaft wirksamen operativen Therapie [15, 25]. Diese besteht aus einer regelmäßigen manuellen Lymphdrainage (zunehmend ergänzt durch eine automatisierte Lymphdrainage durch ein Gerät, der sog. intermittierend-pneumatischen Kompressionstherapie (IPK; [22])) und Kompressionstherapie mittels Flachstrickbestrumpfung (AWMF s2k-Leitlinie: „Diagnostik und Therapie der Lymphödeme“; aktuell in Überarbeitung). Dabei sollte der Behandler bei der Erstdiagnose eines Lymphödem nicht reflexartig einen Kompressionsstrumpf verschreiben. Vielmehr sollte eine komplexe physikalische Entstauungstherapie (KPE) im ambulanten oder auch stationären Setting begonnen werden. Dabei wird das betroffene Körperteil täglich einer Lymphdrainage mit anschließender elastischer Wickelung so lange unterzogen, bis die überschüssige Lymphflüssigkeit weitestgehend entfernt ist. Erst dann sollte eine Flachstrickware (z. B. Flachstrickkompressionsstrumpf) nach Maß angepasst und konsequent getragen werden. Der Strumpf übernimmt dabei in erster Linie die Aufgabe, die Arbeit der vorangegangenen Lymphdrainage zu erhalten. Daher ist die KPE in 2 Phasen aufgeteilt: Die erste ist die Entstauungsphase und wird oft auch in dafür spezialisierten Kliniken durchgeführt, die zweite Phase ist die Erhaltungsphase. Auch hier wird regelmäßig Lymphdrainage durchgeführt, allerdings in deutlich geringerer Frequenz. Teils werden im Rahmen der KPE noch ergänzende Maßnahmen, wie beispielsweise Aquajogging, angeboten. Viele Patienten berichten jedoch, dass die Erhaltungsphase ihrem Namen leider nicht gerecht wird und die in der Phase 1 der KPE erreichte Volumenreduzierung nur wenige Wochen gehalten werden kann. Folglich sind weitere therapeutische Maßnahmen nötig, um die Beschwerden der Betroffenen besser behandeln zu können.

Auch rekonstruktive chirurgische Eingriffe sind auf eine gute konservative Begleittherapie angewiesen. Wie oben beschrieben, kommt es beim unbehandelten Lymphödem zu einer progredienten Schädigung der Lymphbahnen. Neben der Sklerosierung verlieren die Lymphbahnen auch ihre natürliche Propulsion. Soll Lymphe über neu angelegte lymphovenöse Anastomosen abfließen, muss deshalb in den meisten Fällen mittels konservativer Therapie der Lymphfluss unterstützt werden. Das Gleiche gilt für den freien Lymphknotentransfer – hier muss in vielen Fällen den transferierten Lymphknoten die Lymphflüssigkeit mittels konservativer Therapie zugeführt werden. Idealerweise erhalten Betroffene einen rekonstruktiven Eingriff, bevor das Lymphsystem irreversibel geschädigt ist. Dies ist allerdings aus Erfahrung der Autoren, wie oben bereits angedeutet, die Ausnahme und nicht die Regel.

### Operative Therapie

#### Rekonstruktive Verfahren

##### Lymphovenöse Anastomosen – Supermikrochirurgie.

Als am wenigsten invasives Verfahren und „first-line therapy“, entsprechend der Konsensusempfehlung der *Deutschsprachigen Arbeitsgemeinschaft für Mikrochirurgie der peripheren Nerven und Gefäße (DAM)* aus dem Jahre 2019, ist die Anlage von lymphovenösen Anastomosen. Hierbei werden einzelne Lymphkollektoren unter einem Hochleistungsmikroskop mit ableitenden Venolen anastomosiert [18]. Aufgrund der rasanten Entwicklung der letzten Jahre im Bereich der mikrochirurgischen Instrumente, des Nahtmaterials, der prä- und intraoperativen Bildgebung sowie insbesondere der Hochleistungs-Operationsmikroskope können hier zunehmend gut reproduzierbare und dauerhafte Verbesserungen der Symptome erzielt werden [13]. Da es sich bei den Lymphbahnen in der Regel um Strukturen von einer Größe unterhalb eines Millimeters handelt, wurde für diese spezielle Form der Mikrochirurgie von Koshima der Begriff der Supermikrochirurgie geprägt.

**Abb. 5:**
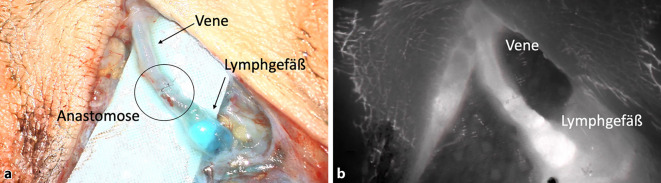
Bild einer angelegten lymphovenösen Anastomose (LVA). **a** Sicht durch das Operationsmikroskop. **b** Sicht durch die integrierte Fluoreszenzkamera (das Fluoreszieren der Vene beweist die Durchgängigkeit der Anastomose)

Nach entsprechender Bildgebung und Markierung der Lymphgefäße auf der Haut („lymphatic mapping“) werden im Rahmen eines operativen Eingriffs ein oder mehrere ausgewählte Lymphgefäße freigelegt. Lymphgefäße, die von distal bis zu den proximalen Lymphknoten durchgängig sind, sollten geschont werden. Um die Lymphgefäße während der Präparation besser auffinden zu können, wird oft Patentblau an die gleichen Stellen wie ICG injiziert. In der Regel wird dann das von distal kommende Lymphgefäß mit einer nach proximal verlaufende Venole unter dem Operationsmikroskop anastomosiert. Das können End-zu-End, End-zu-Seit und weitere Arten von Anastomosen sein. Als Nahtmaterial kommt in der Regel 11.0 zum Einsatz, wobei es sich hierbei um eine extrem kleine Nadel mit einem sehr dünnen Faden handelt. Um die Durchgängigkeit zu überprüfen, ist es von großem Vorteil, ein Hochleistungs-Operationsmikroskop mit integrierter Fluoreszenzbildgebung einzusetzen (Abb. 5). Da bei der Anastomose Blut mit klarer (oder blau gefärbter) Lymphflüssigkeit in Kontakt kommt, ist meist die Färbung von Blut dominant und die Anastomose dadurch nur eingeschränkt beurteilbar. Bei der Fluoreszenzbildgebung wird allerdings das Blut von der mit ICG markierten Lymphflüssigkeit überfärbt, sodass die Anastomose in ihrer Durchgängigkeit sowie die Flussrichtung der Lymphe wesentlich besser beurteilt werden können. Zudem können etwaige Leckagen im Bereich der Anastomose besser visualisiert und in der Folge behoben werden. Bei sehr ausgeprägten Schäden an den Lymphgefäßen kann es jedoch vorkommen, dass der gewählte Lymphkollektor keine der beiden Färbungen aufweist. In diesen Fällen kann man die Anastomose nur klinisch und damit nur eingeschränkt beurteilen.

Neben der Durchgängigkeit der Anastomose ist eine gute Abdichtung auch von essenzieller Bedeutung. Anders als bei klassischen Anastomosen im Bereich der Venen oder Arterien, kommt es hier nicht zu einer meist progredienten Abdichtung kleinerer Leckagen, da im Bereich der Anastomosen Blut nicht oder nur kaum vorhanden ist. Dann fließt die Lymphe größtenteils nicht über die Anastomose ab, sondern leckt in die Umgebung, sodass es im Falle eines gewissen venösen Rückstroms zum Verschluss der Anastomosen kommen kann. Der große Vorteil der LVA besteht in ihrer sehr geringen Invasivität und der Tatsache, dass diese oft bereits noch im Operationssaal eine sichtbare Verbesserung des Lymphabstroms bewirken können. Damit eine lymphovenöse Anastomose an der unteren Extremität erfolgreich sein kann, muss zuvor eine chronisch venöse Insuffizienz (CVI) ausgeschlossen werden. Bei CVI kann es im Stehen sonst zu einer Flussumkehr kommen. Da hier der venöse Druck höher als der im Lymphsystem sein kann, würde die Lymphe nicht über die Vene abfließen können [1].

##### Vaskularisierter Lymphknotentransfer.

**Abb. 6:**
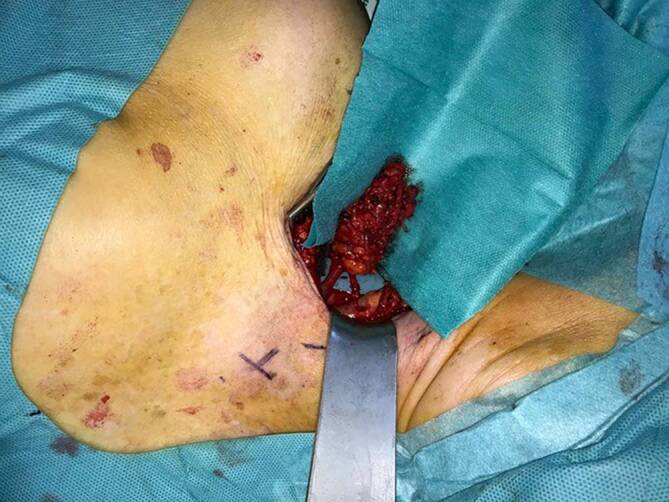
Mikrochirurgisch präpariertes Lymphknotenpaket supraklavikulär

Beim freien vaskularisierten Lymphknotentransfer (VLNT) werden funktionstüchtige Lymphknotenpakete in Form einer freien Lappenplastik von einer gesunden Region in die vom Lymphödem betroffene Region transferiert und mikrochirurgisch angeschlossen. Es gibt eine Reihe von Lokalisationen am Körper, an denen Lymphknoten entnommen werden können, ohne dass es in diesem Bereich dann im Regelfall zu einer Ausbildung eines Lymphödems kommt. Entnahmestellen von Lymphknotenlappenplastiken können beispielsweise supraklavikulär (Abb. 6), thorakal, inguinal, aber auch aus dem Omentum oder dem Mesenterium sein. Beim VLNT werden nicht einzelne Lymphknoten entnommen, sondern Ansammlungen von Lymphknoten, die mitsamt ihrer versorgenden Arterie und Venen gehoben werden. Diese Lymphknotenlappenplastik wird dann an die vom Lymphödem betroffene Region transferiert. Die Arterie und die Venen werden an entsprechende Empfängergefäße unter dem Operationsmikroskop anastomosiert. Im Gegensatz zu dem meist unmittelbaren Wirkeintritt der LVA kann es vorkommen, dass die transplantierten Lymphknoten erst nach bis zu 2 Jahren ihre Wirkung zeigen. Der Wirkmechanismus ist bislang nicht abschließend geklärt. Es werden zwei Theorien diskutiert: die einer Schwammwirkung und die einer zunehmenden Aussprossung afferenter Lymphbahnen. Möglicherweise ist es auch eine Kombination aus beiden Prozessen. Zur Diskussion steht auch die Platzierung der transferierten Lymphknotenplastiken. Dabei wird diskutiert, ob diese weiter proximal oder weiter distal eingesetzt werden sollten.

Trotz größter Sorgfalt besteht natürlich bei der Entnahme von Lymphknotenplastiken die Gefahr eines Entnahmeschadens. Um zu klären, welche Lymphknoten entnommen werden können, kommt das sog. „reverse lymphatic mapping“ zum Einsatz. Will man beispielsweise inguinale Lymphknoten entnehmen, wird ein Radionuklidmarker in die Zehenzwischenräume injiziert. Im Bereich der Leiste können somit diejenigen Lymphknoten mittels Gamma-Sonde detektiert werden, die für den Lymphabstrom des Beins zuständig sind und somit nicht entnommen werden sollten. Zudem wird in den Unterbauch Patentblau injiziert, durch das sich die Lymphknoten in der Leistenregion färben, die als Lymphknotenlappenplastik entnommen werden können. Das Problem eines Hebeschadens in Form eines Lymphödems kann man bei der Entnahme von Lymphknoten aus dem Omentum oder Mesenterium zwar vermeiden, nimmt dabei allerdings die Risiken eines intraabdominellen Eingriffs in Kauf.

Beide Verfahren (LVA und VLNT) haben gemeinsam, dass sie dazu beitragen, Infektraten (Erysipele) signifikant zu reduzieren [12].

#### Resezierende Verfahren

##### Liposuktion.

Ein chronisches Lymphödem ist, wie bereits erwähnt, im Frühstadium ein Flüssigkeitsstau. Es ist *dellbar* und spricht in der Regel gut auf konservative Verfahren an. Im Verlauf kommt es zu einer zunehmenden Akkumulation von Fettgewebe (Abb. 2), das mit einem mehr oder weniger ausgeprägten Grad an Fibrose einhergeht. Lymphbahnen zeigen sich in diesen Stadien in der Bildgebung nur noch vereinzelt. Rekonstruktive mikrochirurgische Eingriffe können hier nur noch bedingt erfolgreich eingesetzt werden. Mittel der Wahl ist die Liposuktion. Bei korrekter Indikationsstellung erübrigt sich hier auch die Frage, ob diese etwaige Lymphbahnen schädigen würde, da man ja gerade erst dann eine Liposuktion in einem Stadium indizieren sollte, in dem keine funktionell relevanten Lymphbahnen mehr vorhanden sind (Stadium III). Mit den Patienten muss im Vorfeld sehr genau besprochen werden, dass auch eine Liposuktion keine dauerhafte Heilung eines Lymphödems erreichen kann. Allerdings können wir heute durch die moderne Bildgebung feststellen, dass sich die Lymphbahnen nach einer Liposuktion (und korrekter Indikationsstellung) bis zu einem gewissen Grad regenerieren können [3]. Die in der abgesaugten Extremität regenerierten Lymphbahnen scheinen teils nach proximal keinen Anschluss zu finden, sodass ein anschließender rekonstruktiv-mikrochirurgischer Eingriff notwendig werden kann. In jedem Fall ist postoperativ eine sehr intensive konservative Therapie nötig und effektiv, um dauerhaft von einer Absaugung zu profitieren.

##### Gewebereduktionsplastiken.

Kommt es durch die zuvor genannten Therapieoptionen nicht zu einer ausreichenden Gewebereduktion, kann die Resektion von überschüssigen Hautlappen, die zu funktionellen Beschwerden führen können, notwendig werden. Teilweise kann auch eine sog. direkte Resektion notwendig sein, die gerade im Bereich von Genitallymphödemen beim Mann große Erleichterung verschafft [23]. Derartige Eingriffe bedürfen einer guten interdisziplinären Zusammenarbeit, um diese komplikationsträchtigen Eingriffe erfolgreich durchführen zu können.

## Fazit für die Praxis

- Das Lymphödem ist eine chronisch progrediente Erkrankung, die mit einer signifikanten Einschränkung der Lebensqualität und sehr oft mit Folgeerkrankungen wie beispielsweise rezidivierenden Eryspielen einhergeht.
- Daher ist die frühzeitige Erkennung des Lymphödems essenziell und eine möglichst frühe Vorstellung bei einem Lymphchirurgen anzustreben, um ein Fortschreiten der Erkrankung zu verhindern bzw. abzuschwächen.
- Die präoperative Diagnostik ist für die Wahl der richtigen Therapie und deren Erfolg unerlässlich.
- Bei primärem Lymphödem und fortgeschrittenen sekundären Lymphödemen ist eine zusätzliche radiologische Diagnostik (MR-Lymphangiographie) sinnvoll.
- Neben einer unverzichtbaren konservativen Therapie führen moderne mikrochirurgische Verfahren in vielen Fällen zu einer erfolgreichen und dauerhaften Symptomlinderung.
